# Long-term intra-individual reproducibility of heart rate dynamics during exercise and recovery in the UK Biobank cohort

**DOI:** 10.1371/journal.pone.0183732

**Published:** 2017-09-05

**Authors:** Michele Orini, Andrew Tinker, Patricia B. Munroe, Pier D. Lambiase

**Affiliations:** 1 Institute of Cardiovascular Science, University College London, London, United Kingdom; 2 Barts Heart Centre, St Bartholomew’s Hospital, London, United Kingdom; 3 William Harvey Research Institute, Barts and The London School of Medicine and Dentistry, Queen Mary University of London, London, United Kingdom; Maastricht University, NETHERLANDS

## Abstract

**Background:**

The heart rate (HR) response to exercise provides useful information about the autonomic function and has prognostic value, but its reproducibility over a long period of time, a critical requirement for using it as a clinical biomarker, is undetermined.

**Aim:**

To determine the intra-individual reproducibility of HR dynamics during sub-maximum exercise and one minute recovery.

**Methods:**

1187 individuals from the Cardio physical fitness assessment test of the UK Biobank repeated a standard exercise stress test twice (recall time 34.2 ± 2.8 months) and were prospectively studied.

**Results:**

821 individuals complied with inclusion criteria for reproducibility analysis, including peak workload differences between assessments ≤10 W. Intra-individual correlation between HR profile during the first and the second assessment was very high and higher than inter-individual correlation (0.92±0.08 vs 0.87±0.11, p<0.01). Intra-individual correlation of indices describing HR dynamics was: ρ = 0.81 for maximum HR during exercise; ρ = 0.71 for minimum HR during recovery; ρ = 0.70 for HR changes during both exercise and recovery; Intra-individual correlation was higher for these indices of HR dynamics than for resting HR (ρ = 0.64). Bland-Altman plots demonstrated good agreement between HR indices estimated during the first and second assessment. A small but consistent bias was registered for all repeated measurements. The intra-individual consistency of abnormal values was about 60–70%.

**Conclusions:**

The HR dynamics during exercise and recovery are reproducible over a period of 3 years, with moderate to strong intra-individual reproducibility of abnormal values.

## Introduction

The resting heart rate (HR) is determined by cardiac autonomic tone, i.e. the net effects of the sympathetic and parasympathetic inputs into the sinus node [1]. Elevated resting HR is a well-established predictor of cardiovascular and all-cause mortality in the general population [2,3]. However its predictive value is thought to be an epiphenomenon of different systemic conditions rather than truly representative of a causal link between HR and adverse outcome [2,3]. Resting HR provides useful information regarding the autonomic tone but does not characterize autonomic reactivity and modulation. The capability of the autonomic system to respond to challenges and stressors can be assessed by measuring changes in HR during and after exercise. Mild exercise is associated with a progressive decrease in parasympathetic activity which is completely abolished at higher workloads when HR acceleration is mainly driven by increased sympathetic activity [4]. The decrease in the HR immediately after cessation of the exercise is thought to be predominately due to parasympathetic activity, with sympathetic withdrawal also contributing in a later phase [5]. Cardiorespiratory fitness and baroreflex sensitivity also modulate the HR response to exercise [4,5]. Furthermore, a small non-autonomic component also contributes to the HR response to exercise [1]. This could include changes in alpha-adrenergic tone, mechano-electric feedback and atrial stretch [6], or temperature changes.

Several studies have demonstrated that indices of heart rate (HR) dynamics during exercise and recovery predict all-cause mortality and cardiac death [7–11] and that the predictive value is independent of systolic function [10], angiographic severity of coronary disease, and exercise capacity [12]. Heart rate indices add incremental prognostic value to advanced diagnostic tools such as myocardial perfusion single-photon emission computerized tomography [13]. The analysis of the HR response to exercise stress test (EST) is useful in screening symptomatic and asymptomatic subjects, and patients with and without arrhythmogenic hereditary syndromes [14,15]. In particular, indices quantifying the HR recovery are recognized as among the most powerful predictors markers for sudden cardiac death [16], with studies showing that they are independent of workload, the presence or absence of myocardial perfusion defects, and changes in heart rate during exercise [8].

Heart rate and other electrophysiological parameters are heritable [17–20], and the identification of possible interactions between genetic traits, HR response to exercise and cardiac risk may be useful for improving the understanding of sudden cardiac death mechanisms and its prevention.

The intra-individual reproducibility of HR dynamics during repeated EST is a critical requirement for potential application as a clinical biomarker and for investigating the genetic architecture of HR response to exercise. A recent study involving 50 individuals has suggested HR recovery after treadmill exercise has short-term (within a month) stability [21], but long term intra-individual reproducibility of HR profile, i.e. the test-retest stability of the time-course of HR, as well as of HR indices during each phase of exercise and recovery has never been assessed in a large cohort.

The aim of this study was to test the hypothesis that indices characterising HR dynamics during repeated EST show long-term intra-individual reproducibility.

The study includes individuals from the UK Biobank data-set who underwent an EST on a stationary bicycle twice over a period of about 3 years, and it demonstrates high intra-individual reproducibility of HR profile and indices of HR response to EST, with important implications for cardiac risk assessment and the understanding of HR dynamics.

## Materials and methods

### Study population and experimental protocol

About 95,000 participants were enrolled in the Cardio physical fitness assessment within the UK Biobank project from 2009 to 2013, a very large and detailed prospective population-based cohort study with over 500,000 participants [22]. Among them, 1187 repeated the EST twice with a recall time equal to 34.3 ± 2.8 months and were included in this study.

The test uses cycle ergometry on a stationary bike (eBike, Firmware v1.7) in conjunction with a 4-lead ECG (CAM-USB 6.5, Cardiosoft v6.51) to record ECGs at rest (15 s pre-test), during graded activity (6 min) and in recovery with hands remaining on the handlebars whilst remaining still and silent (1 min, no cool-down period). The predicted absolute maximum workload was calculated according to a formula given in S1 File, which includes age, height, weight, resting heart rate and sex.

Participants were assigned to two different protocols depending on their physical condition: (1) Cycle for the first 2 minutes at a constant workload, with the pedalling resistance increasing over the last 4 min in steps of 10 W to peak workload defined as 50% of the predicted absolute maximum workload (85% and 85% of all tests during first and second assessment, respectively); (2) Same as previous protocol, but using a peak workload equal to 35% of the predicted absolute maximum workload (15% and 13% of all tests during first and second assessment, respectively). Details are reported in S1 File.

Early termination of the test occurred if the heart rate reached the pre-set maximum HR level of 75% of age-predicted maximum HR (4% and 5% of all tests during first and second assessment, respectively), or in case the subject reported discomfort (less than 0.4% during both assessments).

Specific inclusion criteria for this study are reported in a following section.

This experimental protocol was designed to maximize the information content per unit time and ensure safety. The best test for assessing aerobic capacity, i.e. the maximal oxygen uptake measured during a graded exercise test calibrated to reach exhaustion in about 10 min [23], was considered unfeasible in this large epidemiological study involving individuals with a wide range of abilities, including some with a contra-indication to maximal exercise.

The UK Biobank study was approved by the North West Multi-Centre Research Ethics Committee and all participants provided written informed consent to participate in the UK Biobank study. The study protocol is available online (http://www.ukbiobank.ac.uk/).

### ECG analysis and indices of heart rate profile

**Fig 1:**
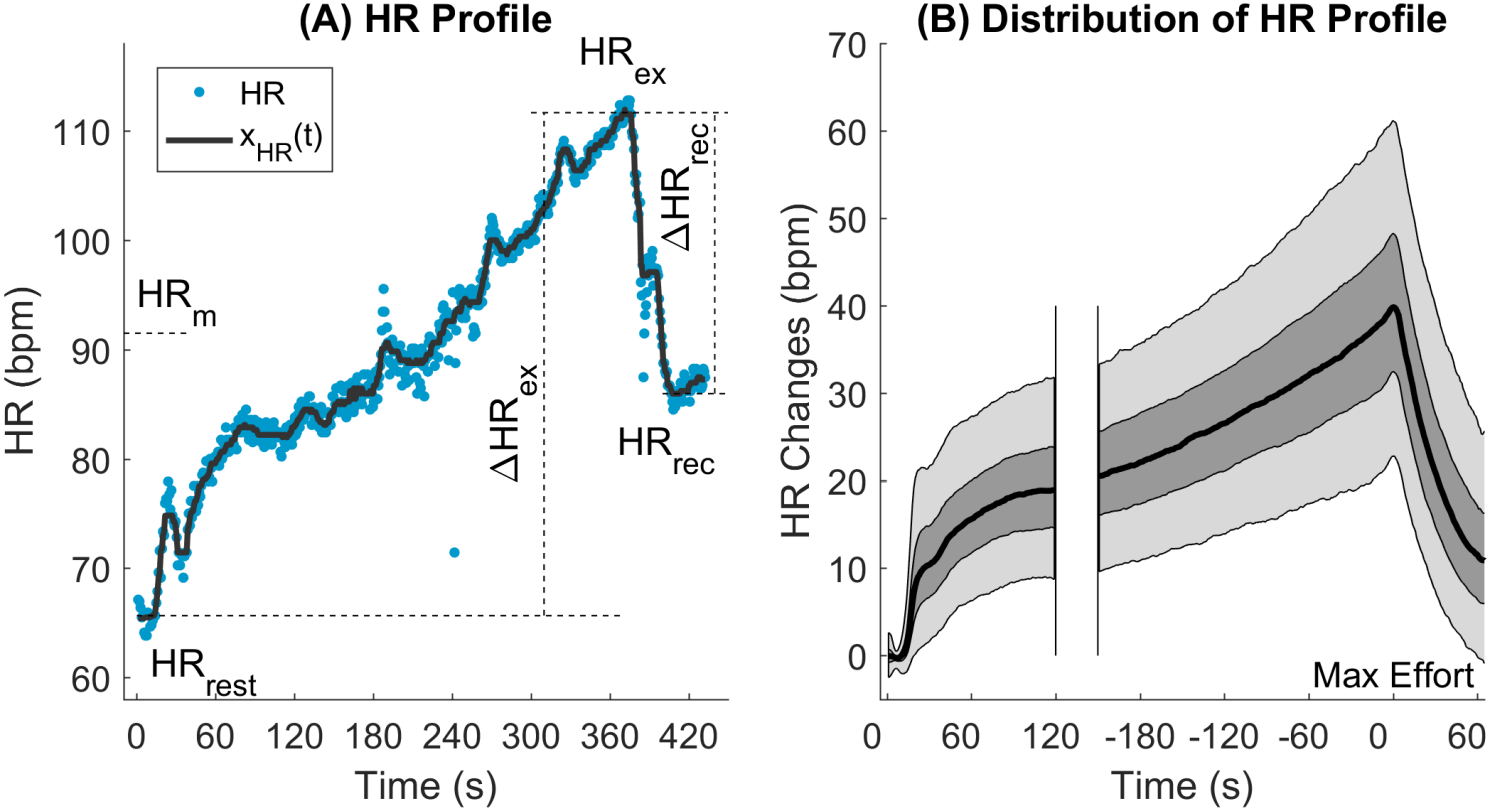
Heart rate profile and indices. A: Definition of heart rate (HR) profile and HR indices during the exercise stress test. The heart rate profile, x_HR_(t) (solid dark line) is a function of time obtained by filtering the instantaneous heart rate (dots) with a median filter 15 beats long. HR_rest_: Mean x_HR_(t) over 15 sec resting pre-test; HR_ex_: Maximum x_HR_(t) during exercise; HR_rec_: minimum x_HR_(t) during recovery; ΔHR_ex_ = HR_ex_ − HR_rest_: HR increase during exercise; ΔHR_rec_ = HR_rec_ − HR_ex_ HR decrease during the 1 min recovery phase. HR_m_: Mean HR during the entire test; B: Distribution of the heart rate profile across all participants. Black solid line, dark and light shadowed areas represent median, 25th-75th percentiles and 5th-95th percentile intervals, respectively.

The electrodes were placed in the right and left antecubital fossae, and left and right wrist and the ECG was sampled at 500 Hz. The RR interval was measured using fully automatic customised algorithms developed in our group during years of experience analysing surface and intra-cardiac ECGs and other cardiovascular signals [24–28]. The HR profile represents the HR dynamics during the EST and is defined as a function of time, x_HR_(t), obtained by filtering the instantaneous HR with a median filter over 15 beats to eliminate respiratory sinus arrhythmia and low frequency oscillations (see solid black line in Fig 1A for a representative example). The following indices were derived from x_HR_(t): Resting heart rate, HR_rest_, defined as the mean x_HR_(t) during the 15 s pre-test period; Maximum HR during exercise (HR_ex_); Minimum HR during 1 min recovery (HR_rec_); HR excursion during exercise, ΔHR_ex_ = HRex − HR_rest_; HR excursion during recovery, ΔHR_rec_ = HR_rec_ -HR_ex_, and mean heart rate during the entire test (HR_m_). Fig 1A provides a graphical description of these parameters.

### Inclusion requirements

Recordings were included in the analysis based on signal quality, x_HR_(t) stability, duration of the tests and differences in the maximum workload assigned during first (EST1) and second (EST2) assessment. Signal quality was assessed calculating the signal to noise ratio (SNR) on a beat-to-beat basis for each ECG lead, and the 25^th^ percentile of the SNR averaged across leads, SNR_25p_, was compared to a threshold value (-3 dB) to include or exclude the recording. The instability of the HR profile was quantified as the standard deviation of the first derivative of x_HR_(t) (expressed in ms), i.e. σ(Δx_RR_) = std([x_HR_(t_i_)_-_ x_HR_(t_i-1_)]^-1^), and HR profiles were considered too unstable if σ(Δx_RR_)>15 ms. HR profile instability, σ(Δx_RR_), is high for x_HR_(t) that show large stepwise changes because of extremely noisy recordings, severe artefacts or a high burden of premature beats, and small when x_HR_(t) varies smoothly according to physiological processes. Participants who reached their pre-set maximum HR level and terminated earlier were included only if the duration of the tests was longer than T>6 minutes. The absolute difference in the maximum workload between EST1 and EST2, |ΔWL|, should not exceed 10 W. However, further subgroup analyses were also performed for different |ΔWL|.

### Statistical analysis

Intra-individual similarity between HR profiles was assessed by calculating the Pearson’s correlation coefficient between the HR profiles, x_HR_(t), during EST1 and EST2. The Pearson’s correlation coefficient provides a measurement of morphological similarity between the time function representing the two HR profiles per participant. The test-retest stability of HR indices was assessed by calculating the coefficient of determination R^2^ (the square of the Pearson’s correlation coefficient) as well as the Spearman’s rank correlation coefficient between HR indices during EST1 and EST2. The Spearman’s rank correlation coefficient is computed on ranks and is robust to outliers. Bland-Altman plots were used to assess agreement between measurements taken during EST1 and EST2.

Since all participants underwent a similar test, high correlation between HR profiles and indices may be due to a general rather than an individual pattern of response to the EST, i.e. HR increases during exercise and decreases during recovery in all individuals. Inter-individual correlation was evaluated by computing the correlation between x_HR_(t) (or HR indices) of different individuals after random permutation of the order of the individuals during EST1 and EST2, i.e. by computing the correlation between X_j,EST1_ and X_k,EST2_ where X represents a given index and j≠k different participants. This procedure was repeated 500 times to assess the inter-individual correlation over a large number of random configurations.

Paired, two-sided Wilcoxon signed rank test was used to assess differences between HR indices during EST1 and EST2. Unpaired tests were performed using the Wilcoxon rank sum test. When appropriate, Bonferroni correction for multiple tests was used as indicated in the text.

Standard box-plots were used to describe data distribution, where central line is the median, the edges of the box are the first (Q1) and third (Q3) quartiles and the whiskers extend to the most extreme data points not considered outliers. Values lower than Q1-1.5*(Q3-Q1) and higher than Q3+1.5*(Q3-Q1) are considered outliers.

## Results

Over the 1887 individuals who repeated the EST twice, 821 (age at EST1 57.8±1 years, 48.5% male) complied with the inclusion criteria (Table 1). The difference in peak workload between EST1 and EST2 was 3.68 ± 3.77 W and the recall time was 34.3 ± 2.8 months. The proportion of individuals assigned to a given maximum workload is reported in Table 1.

**Table 1:** Study population.

**Requirements**	**Unit**	**EST1 & EST2**	
Participants	N (%)	1187 (100)	
T ≥360 s	N (%)	1083 (91)	
SNR > -3dB	N (%)	1083 (91)	
σ(Δx_RR_) < 15 ms	N (%)	1176 (99)	
|ΔWL| ≤ 10 W	N (%)	935 (79)	
Study population	N (%)	821 (69)	
**Characteristics**			
|ΔWL| (mean ± SD)	W	3.68 ± 4.77	
Recall time (mean ± SD)	Months	34.2 ± 2.8	
Gender male	%	48.5	
	**Unit**	**EST1**	**EST2**
Mean Age (mean ± SD)	Years	57.8 ± 7.1	60.6 ± 7.1
40≤WL<60	%	3.5	5.7
60≤WL<80	%	34.2	36.9
80≤WL<100	%	24.2	24.7
100≤WL<120	%	24.0	22.2
120≤WL<Inf	%	13.9	10.2

T: Duration of the EST. SNR: Signal to noise ratio; RRV: HR profile stability; |ΔWL|: Absolute difference in the maximum workload during EST1 and EST2.

### Intra-individual reproducibility of HR profile

The median HR profile, x_HR_(t), showed a sharp increase at the onset of the exercise, then it increased moderately during the first 2 min of cycling at a constant workload, and increased at a higher rate during the last 4 min paralleling the linear increase in workload (Fig 1B). At peak workload, the median HR increased by 40 bpm with respect to the resting HR, with 90% of participants within 22–61 bpm, and it quickly decreased to close to exercise onset level during 1 minute recovery.

**Fig 2:**
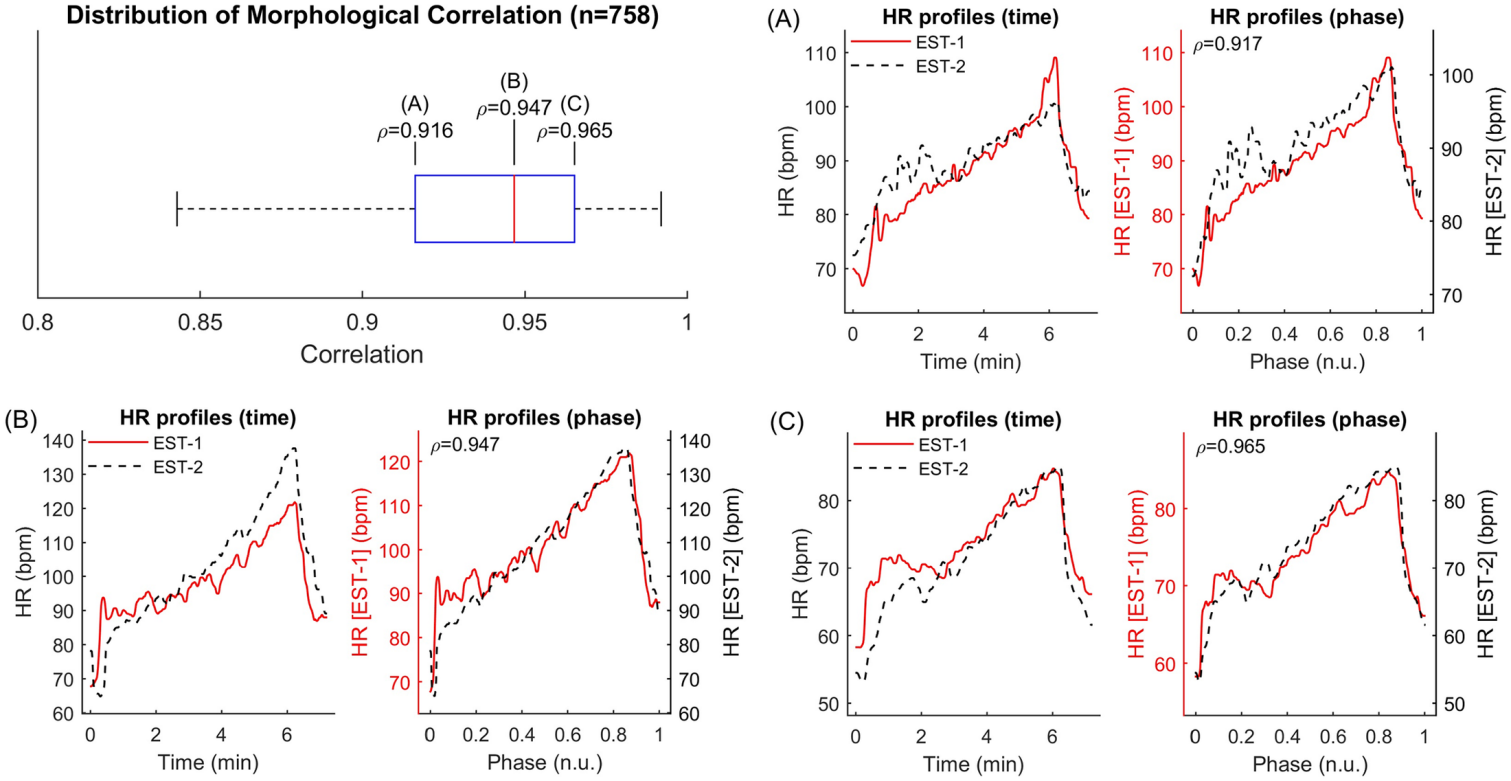
Intra-individual correlation of HR profile. Distribution of the Pearson’s correlation coefficients between heart rate profiles during EST1 and EST2 for all individuals (n = 821). First, second and third quartiles correspond to cases A, B and C, respectively. The HR profiles corresponding to these values are shown in panels (A)-(C). Each panel is composed of two sub-panels, one on the left showing x_HR_(t) against time and using the same vertical axis for EST1 and EST2, and another one on the right showing x_HR_(t) against EST phase (0 and 1 correspond to the beginning and end of each EST) and with different vertical scales adjusted to span the HR range of x_HR_(t) during EST1 and EST2.

HR profile during each phase of EST1 and EST2 followed a very similar pattern even when the baseline HR was different (Fig 2A–2C), with intra-individual correlation equal to 0.947 (0.917–0.965) (median Q1-Q3). The intra-individual correlation between HR profiles was significantly higher than the inter-individual correlation (ρ_pe_ = 0.92 ± 0.08 vs ρ_pe_ = 0.87 ± 0.11, p<0.01, mean ± standard deviation, Bonferroni correction for 500 repeated tests). This supports the hypothesis that the HR response to exercise is subject-specific.

### Intra-individual reproducibility of HR indices

**Fig 3:**
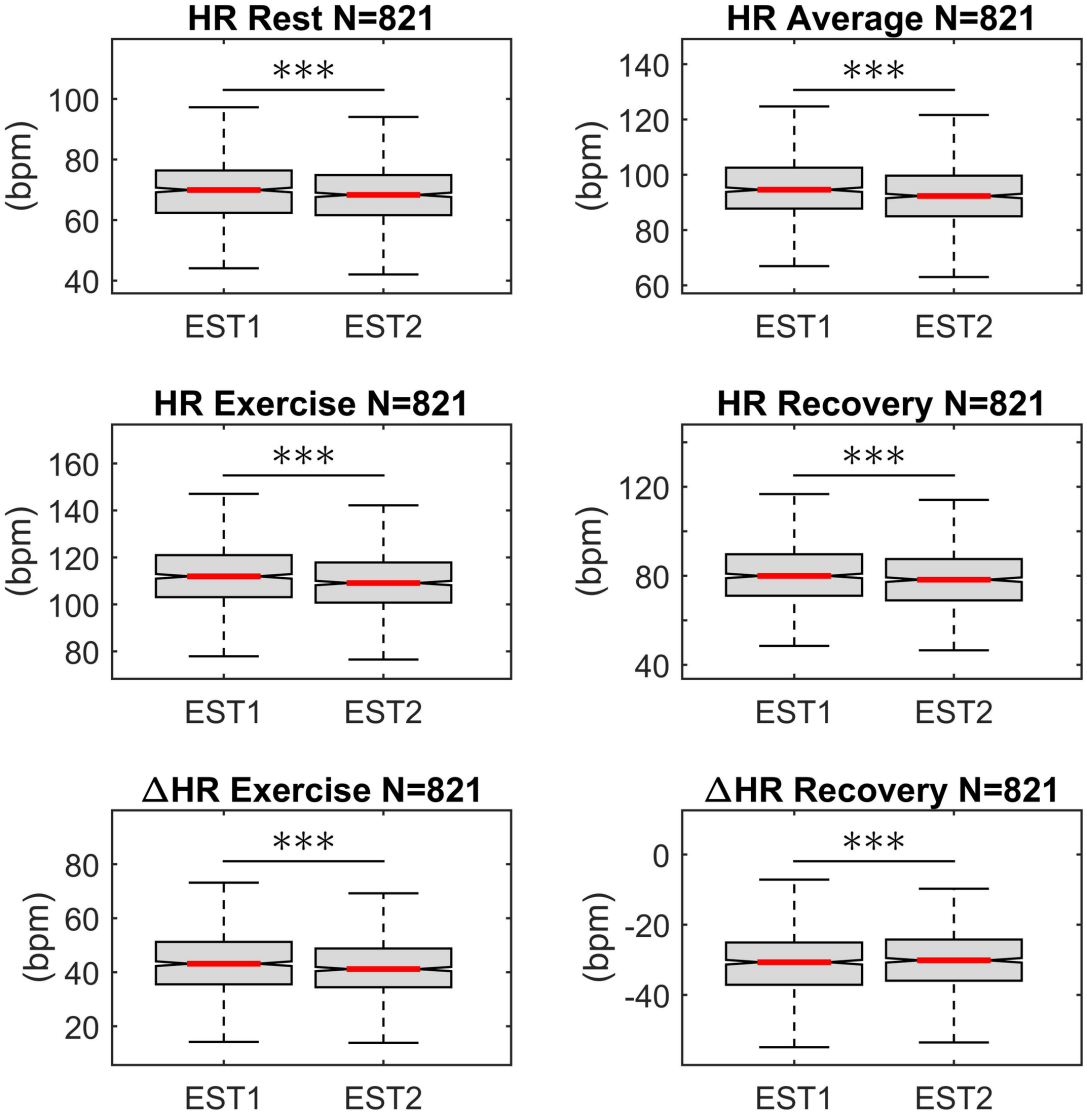
Distribution of heart rate indices. Distribution of heart rate indices during first and second exercise stress test (EST1 and EST2). Every HR index decreased during EST2 with respect to EST1. The decrease was small (<4%) but consistent across individuals. (***) p<5·10^−4^ (Paired, two-sided Wilcoxon signed rank test).

During both EST1 and EST2, inter-subject variability of HR at rest, exercise and recovery was low, with a coefficient of variation (CV) equal to 16%, 13% and 17%, respectively, while CV was equal to 28% and 33% for ΔHR_ex_ and ΔHR_rec_, respectively (Fig 3). There was a subtle but consistent decrease in all indices during EST2 with respect to EST1. The median decrease was lower than 4% but significant on paired sign-rank test for all indices. When comparing HR indices on the subgroup of individuals who during EST1 and EST2 were assigned to a protocol with the same peak workload (|ΔWL| = 0, n = 508), differences in ΔHR_ex_ and ΔHR_rec_ were no longer significant (see S1 Fig). This suggests that both peak workload and age may have contributed to this subtle decrease in the HR indices.

**Fig 4:**
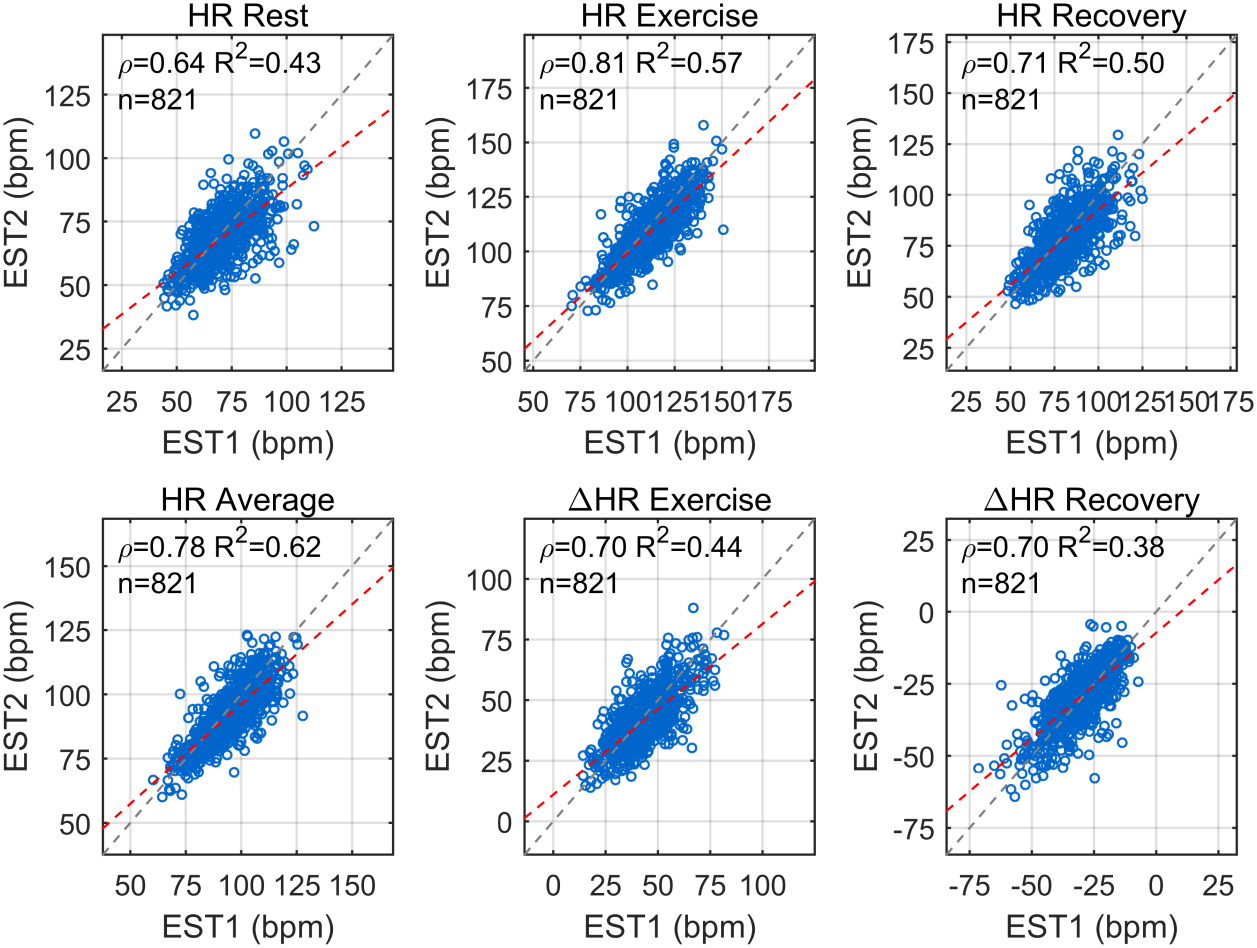
Intra-individual correlation of HR indices. Scatter-plots showing the correlation between HR indices at first (EST1) and second (EST2) assessment. The Spearman’s correlation coefficient, ρ_sp_, and the coefficient of determination, R^2^, quantify the intra-individual correlation for each HR index and are reported in each panel. Dashed grey and red lines represent the identity line and the linear regression line, respectively.

Indices describing the dynamic response to exercise showed high intra-individual correlation (Fig 4). The maximum heart rate, HR_ex_, had the highest correlation (ρ_sp_ = 0.81), followed by HR_m_ (ρ_sp_ = 0.78), HR_rec_ (ρ_sp_ = 0.71), ΔHR_ex_ (ρ_sp_ = 0.70) and ΔHR_ex_ (ρ_sp_ = 0.70). Of note, the correlation for these HR indices was higher than for the resting heart rate, HR_rest_, for which ρ_sp_ = 0.64. For all indices, the slope of the linear regression line was slightly ≤1. Intra-individual correlation was much higher than inter-individual correlation which was almost zero.

**Fig 5:**
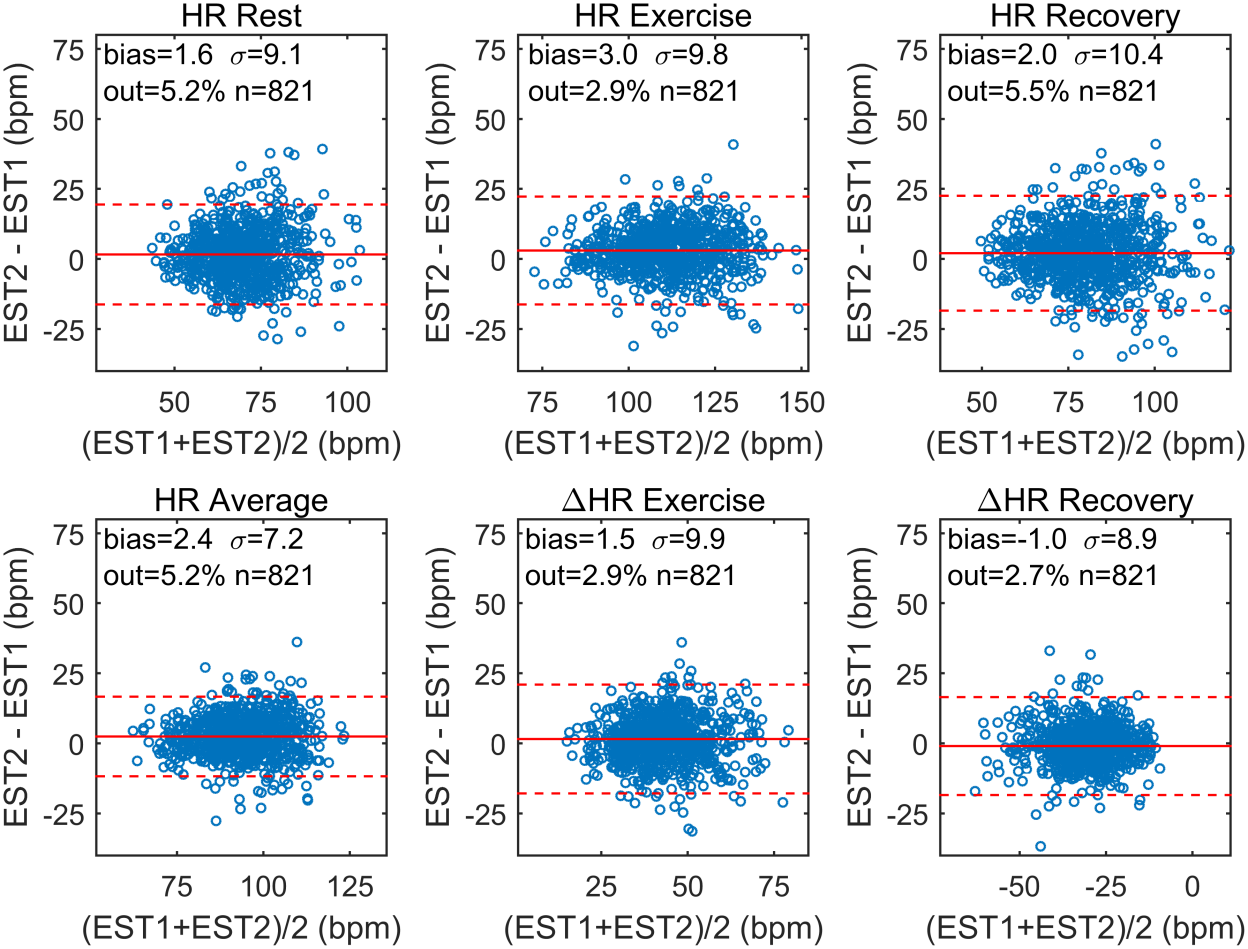
Intra-individual agreement for heart rate indices. For each HR index, Bland-Altman plots were used to assess intra-individual agreement. For each individual, the difference in each index between EST2 and EST1 (x2-x1) is plotted against the mean value (x_1_+x_2_)/2. Each subplot reports the bias, i.e. mean(x_2_-x_1_), the standard deviation of the differences, i.e. σ = std(x_2_-x_1_), the number of individuals outside the limits of agreements (%), and the total number of individuals. The confidence interval (bias ± 2σ, red lines) is reported in dashed lines.

Bland-Altman plots show good agreement between HR indices during EST1 and EST2 (Fig 5). There was a small but significant bias in most of the indices, consistent with the decrease in the HR indices observed in EST2. The bias was constant over the entire range of each index. The variability of (x_2_-x_1_) was relatively small compared to the index mean values, with the ratio between the standard deviation of (x_2_-x_1_) and the mean of (x_1_+x_2_)/2 equal to 8% and 13% for HR_ex_ and HR_rec_ and equal to 23% and -28% for ΔHR_ex_ and ΔHR_rec_, respectively.

Correlation and agreement for the slopes characterizing the rate of HR increase and decrease during exercise and recovery, respectively, (see S2 Fig) were similar to correlation and agreement characterizing HR_ex_ and HR_rec_. This is not surprising since the duration of exercise and recovery in the experimental protocol was the same during both assessments, i.e. equal to 6 and 1 minutes, respectively, for the vast majority of the participants.

### Reproducibility of extreme values

**Fig 6:**
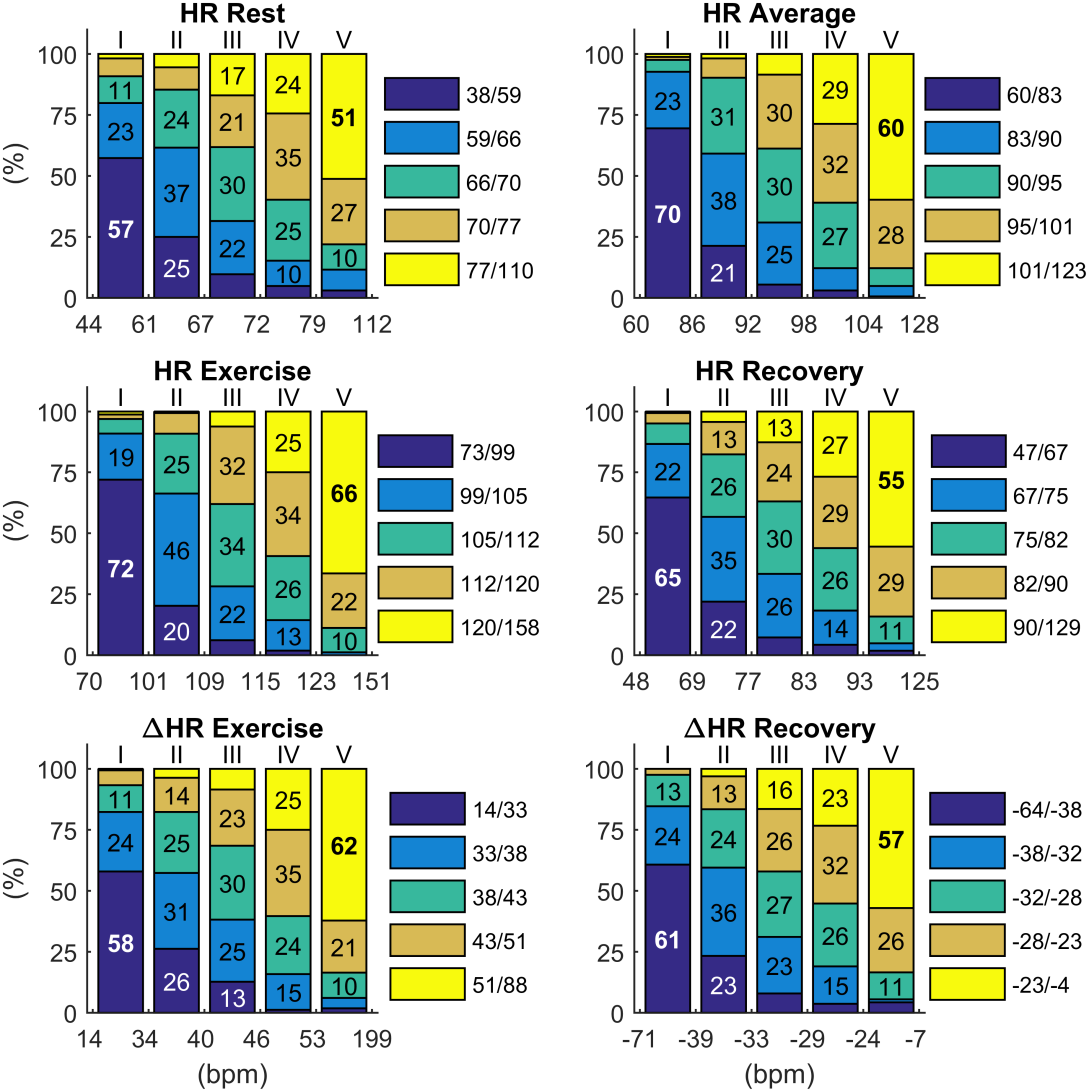
Intra-individual reproducibility of extreme values. Intra-individual reproducibility of abnormality, defined as the probability for an individual who was in a given quantile during EST1 to remain in the same quantile or to change of quintile during EST2. For example, intra-individual reproducibility of extreme values for ΔHR_ex_ (first quintile) and ΔHR_rec_ (fifth quintile) was 58% and 57%, respectively.

The analysis of extreme or abnormal HR indices have been used to identify individuals at risk. Individuals in the extreme quintiles of ΔHR_ex_ and ΔHR_rec_ during EST1 had c. 60% probability of remaining in the same quintile during EST2, and c. 85–90% probability of either remaining in the same quintile or falling in the adjacent one during EST2 (Fig 6). Reproducibility of extreme values for HR indices during EST was slightly higher than for resting HR. The highest reproducibility of extreme values was observed for HR_ex_, HR_m_ and HR_rec_, with 72%, 70% and 65% of individuals remaining in the lowest quintile during both EST1 and EST2. Consistency was higher for individuals in extreme quintiles than for individuals in between the second and fourth quintiles.

### Influence of maximum workload differences

**Fig 7:**
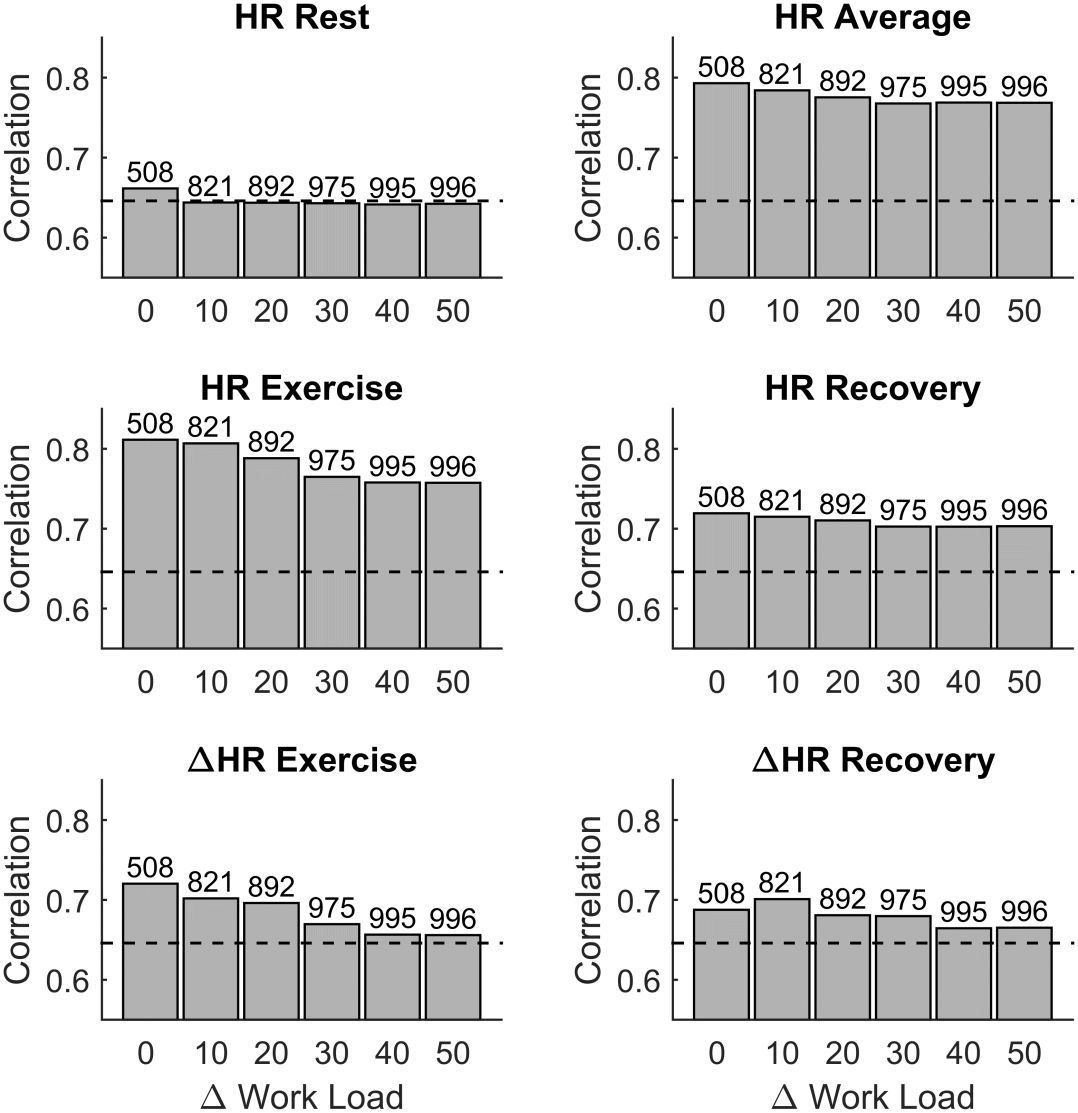
Influence of maximum workload differences on intra-individual correlation. Intra-individual correlation of HR indices was assessed considering individuals who underwent ESTs with maximum work load that did not differ more than |ΔWL|. Bars represent the Spearman’s correlation coefficient. The number of individuals for each group is reported on top of each bar. Horizontal line is the mean Spearman’s correlation coefficient for resting HR.

The results shown so far were obtained analysing data recorded during ESTs for which the difference in peak workload between first and second assessment was |ΔWL|≤10 W. Since the difference in the peak workload may both affect the maximum HR and be an indication of changed physical conditions (for a description of the factors affecting the peak workload please refer to the formula given in S1 File) it is reasonable to hypothesise that |ΔWL| may affect the reproducibility of HR indices. This was confirmed in Fig 7, which shows that |ΔWL| was negatively associated with the intra-individual correlation of indices quantifying HR changes during exercise and recovery, but did not affect the resting HR. Interestingly, the intra-individual correlation of HR indices during exercise and recovery remained higher than intra-individual correlation of resting HR for all |ΔWL|. Please note that more than the 821 participants considered so far were included in this analysis for |ΔWL|>10.

## Discussion

This study includes a large number (more than 800 in most of the analysis) of individuals who repeated a standardized EST on a stationary bicycle and investigates the long-term intra-individual reproducibility of the HR response to exercise. The main results are: (1) The intra-individual correlation between the HR profiles, x_HR_(t), was very high (ρ_pe_>0.92 and ρ_pe_>0.94 in 75% and 50% of participants, respectively, Fig 2) and significantly higher than the inter-subject correlation; (2) Intra-individual correlation for HR indices of exercise and recovery was ≥0.70, and was higher than intra-individual correlation of resting HR (Fig 4). Bland-Altman plots demonstrate that there was a good agreement between indices estimated at EST1 and EST2 (Fig 5); (3) A small but significant bias was registered for all repeated measurements. This reflects the small but significant reduction in HR indices during EST2 with respect to EST1 (Fig 3), which may be due to the effect of aging (mean recall time was about 3 years) on cardiorespiratory fitness and on the peak workload (which slightly decreases with age). (4) The consistency of abnormal values, defined as the probability of an individual of remaining in the same extreme quintile of the distribution of a given index, was about 60–70%. It increased to about 85–90% when consistency was extended to the next quintile.

Overall, these results suggest that the HR response to exercise is subject-specific and it is reproducible with moderate to high degree of similarity over a period of at least 3 years.

### Test-retest stability and clinical implications

The assessment of the HR profile, and in particular of HR recovery, during EST has been shown to be predictive of mortality, both when the test is performed on a treadmill [9–13,29–33] or on a stationary bicycle [7,34,35], both in specific cohorts including symptomatic and asymptomatic patients and in the general population [7].

In a large study based on the entire Cardio physical fitness assessment of the UK Biobank data-set, which includes the data analysed in this paper, a marker of cardiorespiratory fitness based on HR changes was associated with mortality [36]. Although in some studies the HR response to EST was predictive of cardiovascular death and arrhythmic events, most of the studies suggest that HR profile information is more predictive of all-cause mortality [9]. According to guidelines recommendations, an exercise ECG may be considered for cardiovascular risk assessment in intermediate-risk asymptomatic adults [37,38].

However, the intra-individual reproducibility of HR response to EST, which is crucial to determine its reliability, had yet to be determined. Test-retest stability is a fundamental requirement for a biomarker, and consistency of abnormal results over repeated measurements can improve stratification, as demonstrated by the fact that test-retest stability of abnormal response to EST before and after cardiac rehabilitation improves mortality prediction [32,39].

The short-term (over few months) intra-individual reproducibility of HR recovery after treadmill exercise was assessed in few small studies (less than 90 patients), which reported high correlation and good agreement [21,40], but warned about possible insufficient test-retest consistency for abnormality [40]. Our results confirm high correlation and agreement for HR recovery (ΔHR_rec_) in a larger cohort of the general population over a much longer period of time (about 3 years), and show that test-retest consistency for abnormality was around 60%. Similar results were found for HR changes during exercise (ΔHR_ex_) and minimum HR during recovery (HR_rec_), while better test-retest stability for abnormal values was found for peak HR (HR_ex_). Intra-individual reproducibility was higher in exercise parameters than resting HR. However, a variability in test-retest measurements exists and should be taken into consideration for abnormality detection or if a test-retest strategy is used to assess the evolution of mortality risk [32,39]. For example, the standard deviation of the differences in ΔHR_rec_ between EST1 and EST2 was 8.9 bpm (Fig 6).

A mild reduction in the intra-individual correlation of HR indices was observed for increasing differences in peak workload. However, since the peak workload depended on the participant’s physical condition, the negative association between intra-individual correlation of HR indices and differences in peak workload may be in part due to a change in the participant’s physical condition.

Of note, this study reports for the first time a high intra-individual correlation for the shape of the HR profile x_HR_(t), i.e. the continuous and dynamic changes during exercise. This suggests that the evolution of HR during exercise and recovery is subject-specific and may add valuable clinical information to the quantification of HR changes at maximum effort and during recovery. Mathematical methodologies that provide a quantitative assessment of the morphological differences between the time course of two variables, which have been already applied to the analysis of cardiac repolarization dynamics [41,42], may add to or have better prognostic value than the heart rate recovery. In fact, the characterization of the shape of the HR profile during the different phases of an EST may better represent the complex interplay between sympathetic and parasympathetic activation and withdrawal that determines the HR response to exercise [1,4] and be more accurate in revealing autonomic imbalance.

The dynamic HR response to exercise may have a genetic basis. Moderate heritability (32–34%) has been demonstrated for exercise and recovery HR during sub-maximal treadmill test [43,44], while a more recent tween study found higher heritability (65%) for HR recovery after one minute from exhaustion in healthy adolescents [18]. Recent studies have characterized the genetic contribution to resting HR [45] and HR variability [46] and their interaction with mortality, but direct link between HR response to exercise and specific genes has not yet been found. Specific genotype-phenotype interactions revealed by exercise are relevant to determine cardiac risk associated to specific syndromes, such as long QT [15] and catecholaminergic polymorphic ventricular tachycardia [47]. The definition of the genetic architecture of the dynamic response to exercise may be useful to better understand sudden cardiac death mechanisms and improve risk stratification.

This study has limitations. The EST protocol was sub-maximal and relatively short, which is not ideal to assess cardio-respiratory fitness and HR response to exercise. A longer test continued until physical exhaustion was excluded because of the large epidemiological nature of the study including in total about 95,000 individuals. No information regarding the physical conditions of the participants at the time of the two assessments was available to the authors. The analysis of the interaction between intra-individual correlation of HR indices and differences in peak workload (which is determined by, among others, age, resting heart rate and weight) suggests that reproducibility may have increased if the analysis was restrained to participants maintaining similar physical conditions over the three years separating the two assessments. The high intra-individual reproducibility reported in this study despite the lack of a stringent control over the participants’ physical condition reinforces the result of the study.

## Conclusions

In a cohort of more than 800 individuals from the general population, the dynamic changes in the HR and the indices describing the HR response to a standardized exercise stress test on a stationary bicycle show high intra-individual reproducibility and moderate to strong test-retest stability of extreme values over a period of three years. This is an important requirement for risk stratification. Future studies are needed to define the genetic architecture of the dynamic response to exercise and investigate its interaction with cardiac autonomic modulation and adverse outcome including cardiovascular and non-cardiovascular mortality.

## Supporting information

S1 File Exercise stress test protocol description. This file contains a detailed description of the exercise stress test protocol.
(DOCX)

S1 Fig Distribution of heart rate indices for individuals assigned to the same peak workload. Distribution of heart rate indices during first and second exercise stress test (EST1 and EST2). Only individuals assigned to the same peak workload were considered (n = 508). (***) p<5·10^−4^, Paired, two-sided Wilcoxon signed rank test.
(JPG)

S2 Fig Analysis of the slopes characterizing the rate of HR increase and decrease during exercise and recovery, respectively. Slopes α-exercise and α-recovery have been measured calculating the linear regression of x_HR_(t) during exercise and recovery, respectively. Left panels: Distribution of α-exercise and α-recovery during first and second exercise stress test (EST1 and EST2). α-recovery but not α-exercise decreased during EST2 with respect to EST1. (*) p<5·10^−4^ (Paired, two-sided Wilcoxon signed rank test). Middle panels: Scatter-plots showing the correlation between α-exercise and α-recovery at EST1 and EST2. The Spearman’s correlation coefficient, ρ_sp_, and the coefficient of determination, R^2^, quantify the intra-individual correlation are reported in each panel. Dashed grey and red lines represent the identity line and the linear regression line, respectively. Right panels: Bland-Altman plots were used to assess intra-individual agreement. Each subplot reports the bias, i.e. mean(x_2_-x_1_), the standard deviation of the differences, i.e. σ = std(x_2_-x_1_), the number of individuals outside the limits of agreements (%), and the total number of individuals. The confidence interval (bias ± 2σ, red lines) is reported in dashed lines.
(JPG)
