# Correction: Long-term intra-individual reproducibility of heart rate dynamics during exercise and recovery in the UK Biobank cohort

**DOI:** 10.1371/journal.pone.0193039

**Published:** 2018-02-12

**Authors:** Michele Orini, Andrew Tinker, Patricia B. Munroe, Pier D. Lambiase

The following information is omitted from the Funding section: This research has been conducted using the UK Biobank Resource under Application Number 8256 and supported in part by Medical Research Council grant MR/N025083/1.

## References

[1] Orini M, Tinker A, Munroe PB, Lambiase PD (2017) Long-term intra-individual reproducibility of heart rate dynamics during exercise and recovery in the UK Biobank cohort. PLoS ONE 12(9): e0183732 https://doi.org/10.1371/journal.pone.0183732 28873397 10.1371/journal.pone.0183732 PMC5584807

