# Supplementary material for: Long-term intra-individual reproducibility of heart rate dynamics during exercise and recovery in the UK Biobank cohort
Source: PLoS One. 2017 Sep 5;12(9):e0183732. doi: 10.1371/journal.pone.0183732 (PMC5584807; doi:10.1371/journal.pone.0183732)
Supplement: S1 File — This file contains a detailed description of the exercise stress test protocol. (DOCX) [file pone.0183732.s001.docx]

**Workload power calculation**

A complete description of the experimental protocol and acquisition system can be found at:

http://biobank.ctsu.ox.ac.uk/crystal/refer.cgi?id=100229.

For participants in categories 1 and 2, i.e. participants with minimal and small risk, their predicted absolute maximum workload is calculated using the formula:

ABSOLUTE MAXIMUM WORKLOAD =

105.2749 + (-0.0935*AGE) + (-0.0280973*AGE*AGE)+

+ (2.809493*SEX) + (119.0087*HEIGHT) + (0.309456*WEIGHT)+

+ (-2.698067* RHR) + (0.0090985 *RHR*RHR) + (-0.3783405 * AGE * SEX)+

+ (60.72548 *HEIGHT*SEX) + (-0.15016*WEIGHT*SEX) +

+ (-0.3730664*RHR*SEX) + (0.0180811*RHR*AGE)

Where:

- AGE [years]: Age, calculated from date of birth.
- SEX [none]: Factor equal to 0 for females and 1 for males.
- HEIGHT [meters]: Height, taken from Biometric stage.
- WEIGHT [Kg]: Weight, taken from Biometric stage.
- RHR [bpm]: Resting heart-rate, taken as lowest value from BP measurement in Interview

For participants in Category 1, the target-power is 50% of the absolute-max-workload.

For participants in Category 2, the target-power is 35% of the absolute-max-workload.

After the target power has been calculated the participant is assigned to one of the following protocols (see Tables here below) – chosen to be the “hardest” protocol for which the peak power does not exceed the participants target power.

All bicycle protocols consist, in order, of Initial 15 seconds rest (pretest resting ECG) 2 minute phase at constant power Linear increase over 4 minutes from Start to Peak power level Concluded by a 1 minute recovery period.

The ECG measurement is taken throughout the 7 minutes 15 seconds period.

| **Female Participants** | | **Male Participants** | |
| --- | --- | --- | --- |
| Start Power (W) | Peak Power (W) | Start Power (W) | Peak Power (W) |
| 30 | 30 | 40 | 40 |
| 30 | 40 | 40 | 50 |
| 30 | 50 | 40 | 60 |
| 30 | 60 | 40 | 70 |
| 30 | 70 | 40 | 80 |
| 30 | 80 | 40 | 90 |
| 30 | 90 | 40 | 100 |
| 30 | 100 | 40 | 110 |
| 30 | 110 | 40 | 120 |
| 30 | 120 | 40 | 130 |
| 30 | 130 | 40 | 140 |
