# Supplementary figures and images for: Long-term intra-individual reproducibility of heart rate dynamics during exercise and recovery in the UK Biobank cohort
Source: PLoS One. 2017 Sep 5;12(9):e0183732. doi: 10.1371/journal.pone.0183732 (PMC5584807; doi:10.1371/journal.pone.0183732)

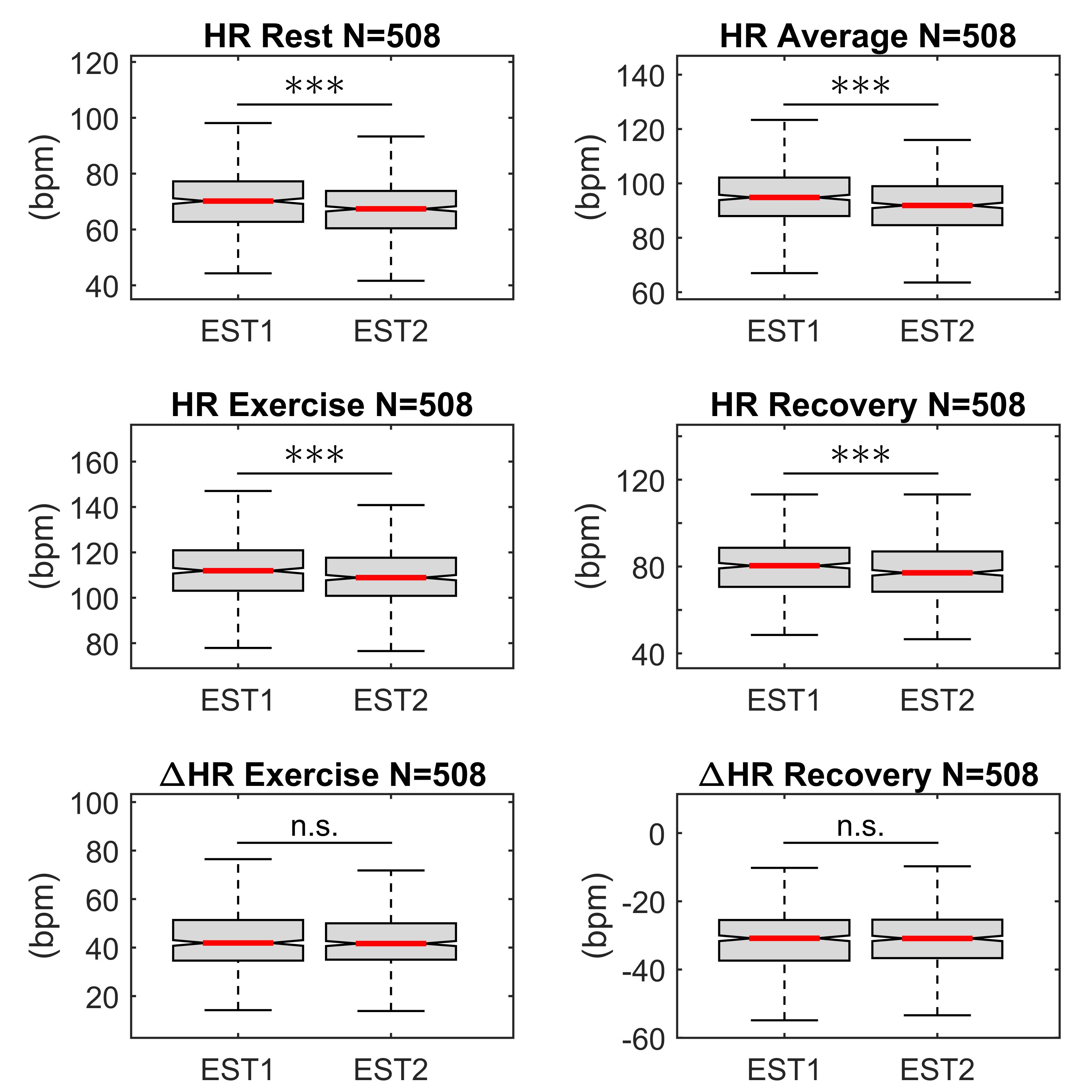

Supplement: S1 Fig — Distribution of heart rate indices during first and second exercise stress test (EST1 and EST2). Only individuals assigned to the same peak workload were considered (n = 508). (***) p<5·10−4, Paired, two-sided Wilcoxon signed rank test. (JPG) [file pone.0183732.s002.jpg]

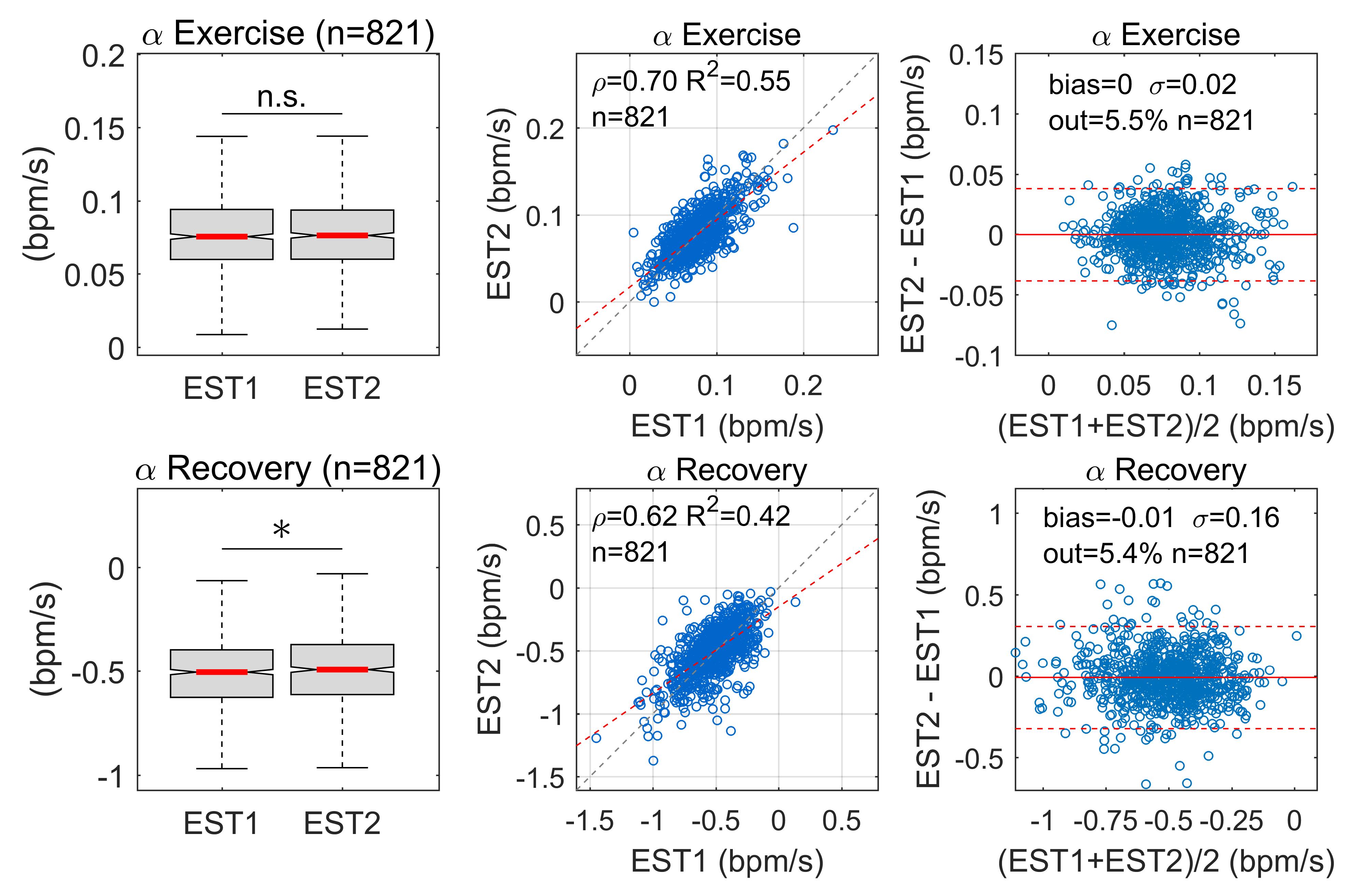

Supplement: S2 Fig — Slopes α-exercise and α-recovery have been measured calculating the linear regression of xHR(t) during exercise and recovery, respectively. Left panels: Distribution of α-exercise and α-recovery during first and second exercise stress test (EST1 and EST2). α-recovery but not α-exercise decreased during EST2 with respect to EST1. (*) p<5·10−4 (Paired, two-sided Wilcoxon signed rank test). Middle panels: Scatter-plots showing the correlation between α-exercise and α-recovery at EST1 and EST2. The Spearman’s correlation coefficient, ρsp, and the coefficient of determination, R2, quantify the intra-individual correlation are reported in each panel. Dashed grey and red lines represent the identity line and the linear regression line, respectively. Right panels: Bland-Altman plots were used to assess intra-individual agreement. Each subplot reports the bias, i.e. mean(x2-x1), the standard deviation of the differences, i.e. σ = std(x2-x1), the number of individuals outside the limits of agreements (%), and the total number of individuals. The confidence interval (bias ± 2σ, red lines) is reported in dashed lines. (JPG) [file pone.0183732.s003.jpg]
